# Influence of Antibiotic Administration on the Urinary Bladder Cancer Early Recurrence Rate

**DOI:** 10.1155/2022/9495920

**Published:** 2022-11-08

**Authors:** Filip Kowalski, Tomasz Drewa, Jan Adamowicz, Jacek Wilamowski, Adam Ostrowski, Magdalena Ostrowska, Krzysztof Kamecki, Marcin Miklas, Michal Szlaga, Witold Mikolajczak, Marta Pokrywczynska, Kajetan Juszczak

**Affiliations:** ^1^Chair of Urology and Andrology, Department of Urology, Collegium Medicum, Nicolaus Copernicus University, Bydgoszcz Torun, Poland; ^2^Collegium Medicum, Nicolaus Copernicus University, Bydgoszcz Torun, Poland; ^3^Department of Urology, F. Lukaszczyk Memorial Oncological Center, Bydgoszcz, Poland; ^4^Department of Urology, 10thMilitary Hospital, Bydgoszcz, Poland; ^5^Department of Urology, M. Curie-Sklodowskiej Memorial Voivodeship Hospital, Zgierz, Poland; ^6^Department of Urology, Nicolaus Copernicus Municipal Hospital, Torun, Poland; ^7^Chair of Urology and Andrology, Department of Regenerative Medicine, Cell and Tissue Bank, Collegium Medicum, Nicolaus Copernicus University, Bydgoszcz Torun, Poland; ^8^Department of Urology, L. Rydygier Memorial Specialist Hospital, Krakow, Poland

## Abstract

Bladder cancer tends to recur, making treatment one of the most expensive in oncology. The limited efficacy and high cost of adjuvant therapies in the treatment of bladder cancer prompt research on new drugs which could replace them. In vitro studies have established that antibiotics can have a cytostatic and cytotoxic effect on urinary bladder cancer cells. The objective of the study was to investigate the influence of antibiotics on the recurrence rate of bladder cancer. In a retrospective study, we analyzed a group of 199 patients with urinary bladder cancer from four urological centers. The study groups consisted of 40 patients who received ciprofloxacin and 83 patients who received beta-lactams as perioperative antimicrobial prophylaxis. The control group included 76 patients who did not get perioperative antimicrobial prophylaxis. The groups were analyzed for risk stratification, degree of malignancy, and size of the primary tumor. The average follow-up time was 24 months. The main focus of the study was to investigate the early recurrence rate of bladder cancer among studied groups, which could correlate with the effectiveness of currently used intravesical instillations. Additionally, cancer's early progression was examined. Regardless of the division used, the highest recurrence rate was found in the ciprofloxacin group. There were no statistical differences in the recurrence rate between patients who received beta-lactams and patients who did not receive any antibiotics. In addition, there were no differences due to the progression rate between the groups. Perioperative antibiotic administration does not influence the early recurrence rate in patients with nonmuscle invasive urothelial bladder cancer.

## 1. Introduction

The characteristic feature of bladder cancer is the tendency to recur [1]. The currently used intravesical adjuvant therapies do not sufficiently limit the recurrence rate of urinary bladder cancer [2]. In addition, patients with urinary bladder cancer and co-existing pyuria had a significantly higher rate of high-grade tumors [3]. Antibiotic prophylaxis before surgical treatment of bladder cancer lowers the risk of infection complications. However, properly monitoring pathogen resistance and not escalating antibiotic use without indications is crucial to guide empirical antibiotic therapy [4].

On the other hand, it is postulated that antibiotics have other properties (including the effect on cancer cells), apart from antibacterial activity. *In vitro* studies on cancer cell lines proved the antitumor potential of several antibiotics [5]. In the study, we investigated ciprofloxacin as an antibiotic with the highest antitumor potential *in vitro* studies [6, 7] and beta-lactams as the most often used antibiotics in perioperative prophylaxis for the treatment of urinary bladder cancer [8].

## 2. Aim

The aim of this study was to evaluate the recurrence rate of nonmuscle invasive bladder cancer in patients undergoing transurethral resection of bladder tumors, depending on the usage of antibiotic prophylaxis. Patients who were given ciprofloxacin were assigned to interventional group number 1, while those obtaining beta-lactams were assigned to interventional group number 2 and patients without antibiotics were assigned to the control group.

### 2.1. Materials and Methods

**Figure 1:**
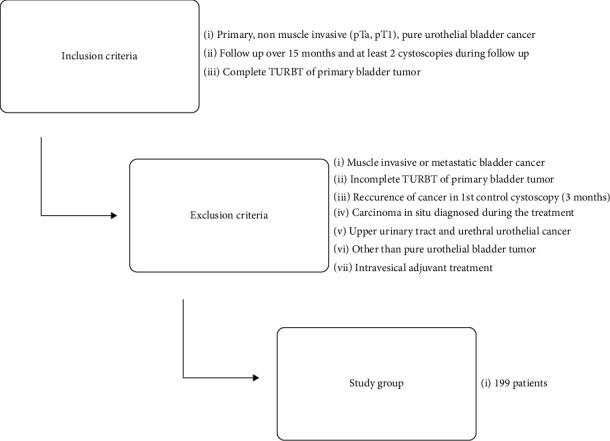
The flowchart of patient selection.

In the retrospect, the study patients came from four urological centers. After application of the inclusion and exclusion criteria, 199 patients treated in the years 2009–2016 were qualified for the study (86 patients from Municipal Hospital in Torun, 61 patients from Oncological Center in Bydgoszcz, 36 patients from 10^th^ Military Hospital in Bydgoszcz, and 16 patients from Voivodeship Hospital in Zgierz). Patients were diagnosed with cystoscopy and primary nonmuscle invasive urothelial bladder cancer was confirmed by transurethral resection of bladder tumor (TURBT) (Figure 1).

The study groups consisted of 40 patients who received ciprofloxacin and 83 patients who received beta-lactams as perioperative antimicrobial prophylaxis (Table 1). The beta-lactams group included patients who received amoxicillin with clavulanic acid, cefuroxime, or cephazolin (Table 1). All antibiotics were given intravenously, first dose 20–30 minutes before TURBT and the subsequent doses after 8–12 hours if there was a high risk of infection or UTI before TURBT. The antibiotic choice was made upon local microbiological recommendations based on previous urine culture results. The high risk of infection was defined as pyuria before TURBT and large tumor mass, which was related to the longer operation time. All patients received only one of the mentioned antibiotics. Patients with more than one antibiotic during the treatment and receiving antibiotics before TURBT were excluded from the study. The control group included 76 patients who did not get peri-operative antimicrobial prophylaxis. Patients who received intravesical instillations with chemotherapy in any form (single immediate instillation or maintained scheme) were excluded. The lack of intravesical treatment in Polish urological departments relates to administrative problems such as the unavailability of drugs and the lack of adequate infrastructure for the administration of chemotherapy. Patients with recurrence in the first control cystoscopy (3 months after TURBT) were excluded because it could indicate incomplete primary resection.

**Figure 2:**
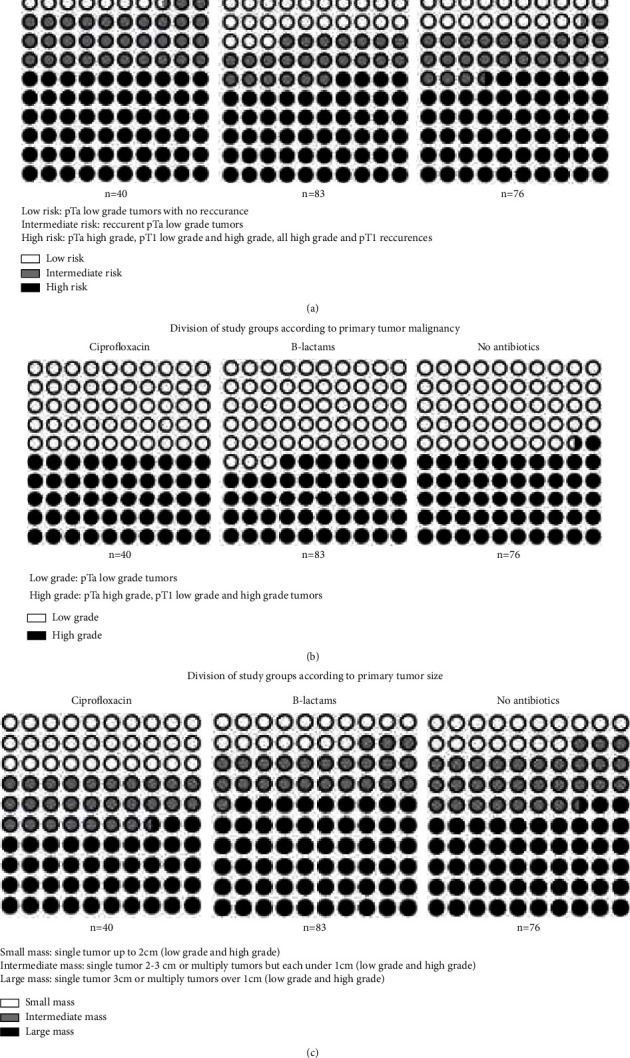
Additional divisions of patients according to (a) Division of study groups according to EAU risk stratification. (b) Division of study groups according to primary tumor malignancy. (c) Division of study groups according to primary tumor size.

For a better investigation of the effects of antibiotics on urinary bladder cancer, additional divisions of the study group were introduced. According to EAU recommendations, groups were analyzed for risk stratification, which corresponded to the course of cancer [28] (Figure 2(a)). Other divisions referred to the influence of antibiotics on the primary tumor. Patients were divided according to the degree of malignancy (Figure 2(b)) and size of the primary tumor (Figure 2(c)). Follow-up was applied according to current EAU recommendations [8]. The basic follow-up tool was cystoscopy. The average time of the follow-up was 24 months (Table 2). Every patient underwent minimum 2 control cystoscopies (on average 3 cystoscopies/patient) (Table 2). The first cystoscopy was done three months after TURBT, the second 12 months after TURBT, and then every 6–12 months. Additionally, every patient during follow-up at least once underwent computer tomography of the abdomen and pelvis floor (uroCT protocol), chest X-ray at least twice, and urinary tract ultrasound during every ambulatory visit (on average, three urinary tract ultrasound examinations/patient). Urine cytology was done among patients with high-grade tumors. Detailed group characteristics are presented in Table 3. Patients who were administered ciprofloxacin had slightly more common pT1 high-grade disease, which corresponded with a more aggressive cancer path, while initially, there were no significant statical differences in cancer advancement (primary tumor malignancy) (Table 3).

Progression was defined as any recurrence with a deeper infiltration and/or a higher grade of malignancy. Statistical analyses were carried out using the IBM SPSS Statistics 25 package. It was used to analyze basic descriptive statistics together with the Kolmogorov–Smirnov test, Mann–Whitney U tests, nonparametric analysis of variance Kruskal–Wallis, χ^2^ tests, and Fisher's exact tests. The recurrence rate was analyzed in Kruskal–Wallis variance with post-hoc analysis using the Dunn–Sidak test with the Bonferroni correction for multiple comparisons. Recurrence rates in particular divisions were analyzed in χ^2^ and Fisher's exact tests. The threshold *α* = 0.05 was considered a statistical significance level. Approval of Ethics Committee: Collegium Medicum Ethics Committee in Bydgoszcz Approval No. KB433/2018.

## 3. Results

**Figure 3:**
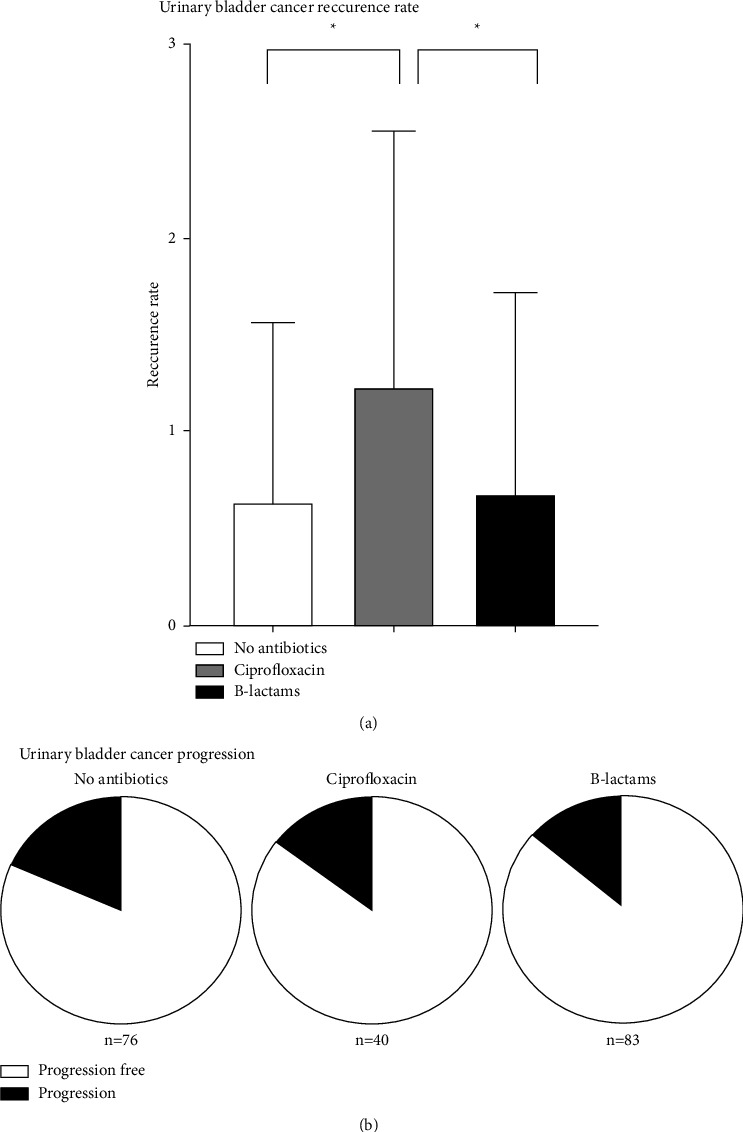
Recurrence and progression rate–general analysis (without additional divisions): ^*∗*^<0.05; ^*∗∗*^<0.01; ^*∗∗∗*^<0.001

In the general analysis. a statistically significant difference was observed in the ciprofloxacin group, patients who received ciprofloxacin experienced more recurrences than patients who did not receive antibiotics (*p*=0.02) or who received beta-lactam antibiotics (*p*=0.017) (Figure 3(a)). When comparing the group of patients not treated with antibiotics and those treated with beta-lactam antibiotics, no statistically significant differences were found in relation to the frequency of cancer recurrence (Figure 3(a)). There were no statistically significant differences in the progression rate. Assuming that antibiotics like anthracyclines should not influence progression rate extended analysis in relation to progression was abandoned (Figure 3(b)).

**Figure 4:**
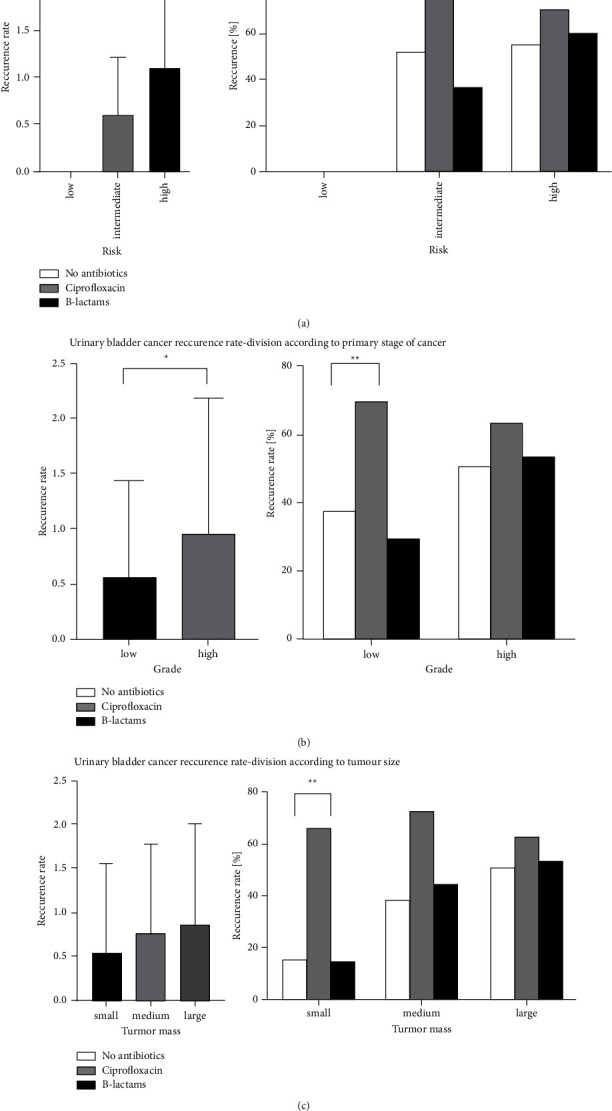
Recurrence and progression rate–analysis of patients in additional divisions. ^*∗*^<0.05; ^*∗∗*^<0.01; ^*∗∗∗*^<0.001.

According to the EAU risk stratification, the recurrence rate was related to the grouping of the applied breakdown. Thus, an apparent statistical difference was demonstrated, in which the frequency of recurrences increased, being the highest in the high-risk group (Figure 4(a)). Furthermore, statistical analysis of the studied groups showed a statistical tendency (*p*=0.083) of more frequent cancer recurrences in the intermediate-risk group among patients who received ciprofloxacin. However, it was not statistically significant (Figure 4(a)).

### 3.1. Division of Study Groups According to Primary Tumor Malignancy

By assessing the study groups concerning the degree of malignancy of the primary tumor, a statistical trend showed a higher frequency of recurrences in patients with the high-risk primary disease (Figure 4(b)). Patients from the low-risk group who received ciprofloxacin experienced a statistically significantly more frequent recurrence than patients who did not receive any antibiotics (*p*=0.009). There was no stastical difference between patients who received beta-lactams and did not receive any antibiotics (Figure 4(b)).

### 3.2. Division of Study Groups According to Primary Tumor Size

The statistical trend of more frequent recurrences with initially large tumor mass was confirmed in this division (Figure 4(c)). It has not been confirmed that the use of any of the antibiotics reduces the frequency of recurrences. In the division by tumor mass, there was again a statistically more frequent recurrence rate among patients treated with ciprofloxacin in comparison to patients who did not receive any antibiotics (*p*=0.010) and there was no statistical difference among patients who received beta-lactams as those who did not receive any antibiotics (Figure 4(c)).

## 4. Discussion

### 4.1. Analysis of Patients Included in the Study

Inclusion and exclusion criteria were used to select patients who could benefit from the use of antibiotics in anticancer therapy. Assuming that antibiotics have similar efficacy to anthracyclines (including doxorubicin), such a group of patients should be patients with low-risk nonmuscle invasive urothelial bladder carcinoma. Patients who received ciprofloxacin and beta-lactams in perioperative prophylaxis were selected for the study. Patients who received ciprofloxacin were selected due to the high antitumor potential of ciprofloxacin proved *in vitro* studies [9–12]. Patients who received beta-lactams were selected due to the widespread use of these antibiotics in perioperative prophylaxis and antitumor potential proved *in vitro* studies [13–16]. The main aim of the study was to examine the early recurrence rate, which could be the best reference to effectiveness in relation to anthracyclines as their most significant benefit is the limitation of time to first recurrence [17].

### 4.2. Postulated Mechanism of the Action

The antibacterial activity of ciprofloxacin is based on the inhibition of type II topoisomerase [9]. Ciprofloxacin, combined with topoisomerase II and DNA strands, impairs DNA replication [5]. The inhibition of mitochondrial DNA synthesis leads to the depletion of intracellular ATP resources and consequently to the arrest of the cell cycle in the G2 phase, which initiates p53-dependent apoptosis [7]. In eucaryotic cells, the administration of ciprofloxacin causes an increase in ATM kinase serum concentration, which is responsible for DNA repair, which suggests that ciprofloxacin can influence eucaryotic cells, including cancer cells [15]. Anticancer activity of ciprofloxacin is believed to relate to the inhibition of topoisomerase II, which leads to S/G2 – M cell cycle arrest and apoptosis of cancer cells [7]. Such an activity is shown by doxorubicin, a recognized drug in the treatment of urinary bladder cancer [9]. In vitro studies have shown that depending on the dose used and the incubation time, ciprofloxacin may have a cytostatic or cytotoxic effect on neoplastic cells of colorectal cancer [10, 11], lung cancer [10], and urinary bladder cancer [10–12]. It has been shown that the concentration of the drug in the range of 50–100 *μ*g/mL inhibits the division of neoplastic cells and the concentration above 400 *μ*g/mL causes apoptosis of neoplastic cells. The optimal incubation time was 24 hours. The antitumor activity catalyst was the medium's pH below 5.5 [11]. The concentration of ciprofloxacin in urine 1-2 hours after oral administration of the drug in a dose of 1 pill of 500 mg averages 400–500 *μ*g/mL, gradually decreasing to the value of about 100 *μ*g/mL 6 hours after administration and 25 *μ*g/mL 12 hours after administration [12, 18]. Intravenous administration of ciprofloxacin achieves a similar drug concentration in urine faster and with a lower drug dose [19].

All beta-lactams have the same antibacterial mechanism based on inhibiting the bacterial cell wall synthesis by inactivating transpeptidases, which results in disturbances in muramine production [13]. It has been suggested that new generations of beta-lactams can affect cancer cells without damaging normal eukaryotic cells [14]. In *in vitro* studies of n-thiol beta-lactams, tumor cells underwent apoptosis by DNA damage induced by caspase 3 and 7 induction. This resulted in cell protein degradation and cell cycle arrest in the G1-S phase [15]. The cytotoxic and cytostatic effect of beta-lactams has been proven for breast cancer cells, prostate cancer, head and neck, lungs, and hematopoietic system [13, 15]. Beta-lactams in the concentration of 50 *μ*g/mL caused apoptosis of the abovementioned neoplastic cells within 48 hours of the optimal incubation time [16]. Potentially, beta-lactams administered 15–30 minutes before transurethral electroresection (TURBT) should reach a concentration having antitumor properties for about 5-6 hours [20].

### 4.3. Analysis of the Results

Regardless of the division used, the highest recurrence rate was found in the ciprofloxacin group. Ciprofloxacin was used in patients who were allergic to beta-lactams. The significantly worse treatment results in the group with ciprofloxacin could be explained by the fact that the follow-up was the longest in this group. On average. the follow-up was four months longer in the ciprofloxacin group than in the other two groups. Despite different follow-up times in the ciprofloxacin group, it should be noted that antibiotics with the highest antitumor potential *in vitro* studies showed the worst results.

No statistical differences existed among patients with high-grade bladder tumors and large tumor mass. This may suggest the lack of effect of treatment with alternative chemotherapy methods when malignancy stage and tumor size are increased. Similar conclusions were made by Elsawy and co-authors, who studied the effect of intravesical Epirubicin postoperative instillation among patients (236 patients) with moderate and high-risk nonmuscle invasive urinary bladder cancer [21]. Moreover, patients from both antibiotic groups did not experience any benefit due to early recurrence, which does not correlate with the reduction of 35% recurrence rate among patients with pTa-pT1 bladder cancer treated by intravesical instillation, which was proved in the randomized trial concerning 2278 patients by Sylvester and co-authors [17].

It should be remembered that in the *in vitro* studies conducted so far, the antitumor effect of antibiotics on cancer cells has been confirmed with long-term incubation of cancer cells (minimum 24 hours) and with a constantly high concentration of the antibiotic [10–14] and that is why the route of administration of the antibiotic is worth considering. No effect of antibiotics given intravenous on bladder cancer cells can be caused by too low concentration of antibiotics and too short time of exposition of cancer cells to antibiotics. Intravesical delivery drugs are more efficient in the adjuvant treatment of nonmuscle invasive bladder cancer, which was proved by D'Ancona and co-authors who compared BCG delivered oral and intravesical; patients who received BCG intravesical had 21% fewer recurrences [22].

However, in complications related to chemotherapeutics and limitations in their availability in some urological departments, for example, in Poland, there are problems due to the lack of adequate infrastructure. explain the sense of looking for new. more available drugs. That is why it is worth considering comparing intravesical instillations of antibiotics with currently used intravesical chemotherapeutic agents. Just urinary bladder irrigation after TURBT has a beneficial effect on reducing the number of recurrences, which was proved by Grivas and co-authors [23]. Therefore, new more accurate methods of tumor resection (en-block) combined with widely available adjuvant treatment can significantly improve treatment outcomes. However, we should remember about growing antibiotic resistance and the specific phenomenon of antibiotics, which can lower the effectiveness of BCG therapy when used chronic what proved Pak and co-authors [24], that is why new studies must be carefully designed.

### 4.4. Limitations of the Study

Retrospective, multicenter, and nonrandomized character of the study caused differences in crucial points such as periods of antibiotics delivery which could make differences in drug concentration in urine. In the study, it was impossible to obtain a proper comparison in terms of cancer progression and late recurrences due to the insufficient follow-up time (average follow-up was 24 months), as well the time of follow-up differed among the groups, which could influence results. The important limitation of the study is the way of drug delivery. Intravenous way of antibiotic dosage may be insufficient to obtain proper urine concentration in adequate time; hence, it would be necessary to project a prospective randomized study with intravesical antibiotics delivery.

## 5. Conclusions

The perioperative antimicrobial prophylaxis given intravenously was of no benefit in relation to the number of early recurrences among patients treated because of primary nonmuscle invasive urothelial bladder cancer.

## Figures and Tables

**Table 1:** Antibiotic use and infectious complications in studies groups.

Antibiotics	Number of patients	Number of doses	Average doses per patient	Dose of the antibiotic	Patients with concomitant UTI
*Beta-lactams*	83	162	1.9/patient		6
(i) Amoxicillin and clavulonic acid	12	71	5.9/patient	1.2 g.i.v.	4
(ii) Cefuroxime	6	19	3.2/patient	1.5 g.i.v.	2
(iii) Cephazolin	65	72	1.1/patient	1 g.i.v.	0
*Ciprofloxacin*	40	232	5.8/patient	0.2 g.i.v.	4

**Table 2:** Follow-up in studies groups.

	Number of patients	Average follow-up time (months)	Number of patients with follow-up time over 18 months	The average number of cystoscopies during follow-up
*Beta-lactams*	83	24 [12–36]	63 [76%]	3 [2–6]
(i) Amoxicillin and clavulanic acid	12	25 [12–36]	8 [67%]	3 [2–6]
(ii) Cefuroxime	6	26 [12–36]	4 [67%]	2 [2–5]
(iii) Cephazolin	65	24 [12–36]	51 [78%]	3 [2–6]
*Ciprofloxacin*	40	24 [12–36]	26 [65%]	3 [2–6]
*No antibiotics*	76	24 [12–36]	53 [70%]	3 [2–6]

**Table 3:** Patients group characteristics depending on the prophylaxis method.

	Beta-lactams	Ciprofloxacin	No antibiotics	(*p* beta-lactam vs no antibiotics/*p* ciprofloxacin vs. no antibiotics)
Number of patients	83	40	76	—/—
Male/Female %	68.7%/31.3%	87.5%/22.5%	76.3%/23.7%	ns/ns
*Percentage of patients in subgroups*				
(i) TaLG	53.0%	47.5%	48.7%	ns/ns
(ii) TaHG	4.8%	5.0%	1.3%	ns/ns
(iii) T1LG	27.7%	10.0%	35.5%	ns/0,03
(iv) T1HG	14.5%	37.5%	14.5%	ns/0,005
Age	62.8	66.8	66.7	0,03/ns
Percentage of patients with a single tumor	68.7%	75.0%	72.4%	ns/ns
*Primary tumor malignancy*				
(i) Low grade	75.0%	82.6%	70.3%	ns/ns
(ii) High grade	25.0%	17.4%	29.7%	ns/ns
*Stratification of patients according to EAU risk groups*				
(i) Low	22.9%	7.5%	18.4%	ns/ns
(ii) Intermediate	22.9%	32.5%	25.0%	ns/ns
(iii) High	54.2%	60.0%	56.6%	ns/ns

## Data Availability

The data used to support the findings of this study are available from the corresponding author upon request.
